# Growth dynamics and light scattering of gold nanoparticles in situ synthesized at high concentration in thin polymer films

**DOI:** 10.3762/bjnano.10.172

**Published:** 2019-08-23

**Authors:** Corentin Guyot, Philippe Vandestrick, Ingrid Marenne, Olivier Deparis, Michel Voué

**Affiliations:** 1 University of Mons, Physics of Materials and Optics, Research Institute for Materials Science and Engineering, Mons, Belgium; 2 AGC Glass Europe - Technovation Centre, Gosselies, Belgium; 3 University of Namur, Physics Department, Namur, Belgium

**Keywords:** gold, imaging ellipsometry, metal nanoparticles, plasmonic nanocomposite, polymer films

## Abstract

**Background:** Numerous optical applications of nano-objects require a dispersion of the nanoparticles in a dielectric matrix. In order to achieve high particle concentrations, one can, as an alternative, directly grow the particles in a polymer or an inorganic film by, e.g., thermal annealing.

**Results:** Simple laser reflection experiments showed that this growth process induced light scattering at the film/air interface. We report on this phenomenon, considering the growth dynamics of gold nanoparticles in a polymer film. The scattering of light was studied by measuring the bi-directional reflection distribution function. In parallel with the observation of enhanced scattering, imaging ellipsometry in dynamics mode showed that local values of the ellipsometric angles Ψ and Δ were strongly modified by the annealing process.

**Conclusion:** A diffraction pattern corresponding to local modifications of the optical properties of the film gradually appeared, which turned out to be the signature of the growth of the Au nanoparticles. Moreover, the monitoring of the statistical distribution of the ellipsometric angles during annealing helped evidencing two regimes in the dynamics of the nanoparticle growth and in the optical response of the nanocomposite.

## Introduction

Over the last 20 years, numerous studies were carried out to investigate the optical properties of plasmonic nanocomposite materials from experimental, theoretical as well as numerical points of view [1–2]. Metal nanoparticles (NPs) play a central role in the development of nanotechnology-based optical devices. Gold nanoparticles (AuNPs) are used in spectrally selective coatings to block solar infrared radiation [3–4], in random lasers [5–6], in non-linear optical applications [7–9] and in sensors or bio-medical diagnostics [10–12]. More recently, nanocomposites containing AuNPs received even more attention due to their saturable absorption. Indeed, plasmonic nanocomposites can be used for constructing passively Q-switched or mode-locked lasers [13–15]. This type of lasers has numerous applications in telecommunications, medicine, material processing and optical fiber sensing [16–17]. Moreover, AuNPs are good candidates for preparing passive Q-switch films because they have large third-order nonlinearity, ultrafast time response (about picoseconds) and saturable absorption behavior [18–19]. However, light scattering at the surface of the device could be an important drawback for this application.

**Figure 1:**
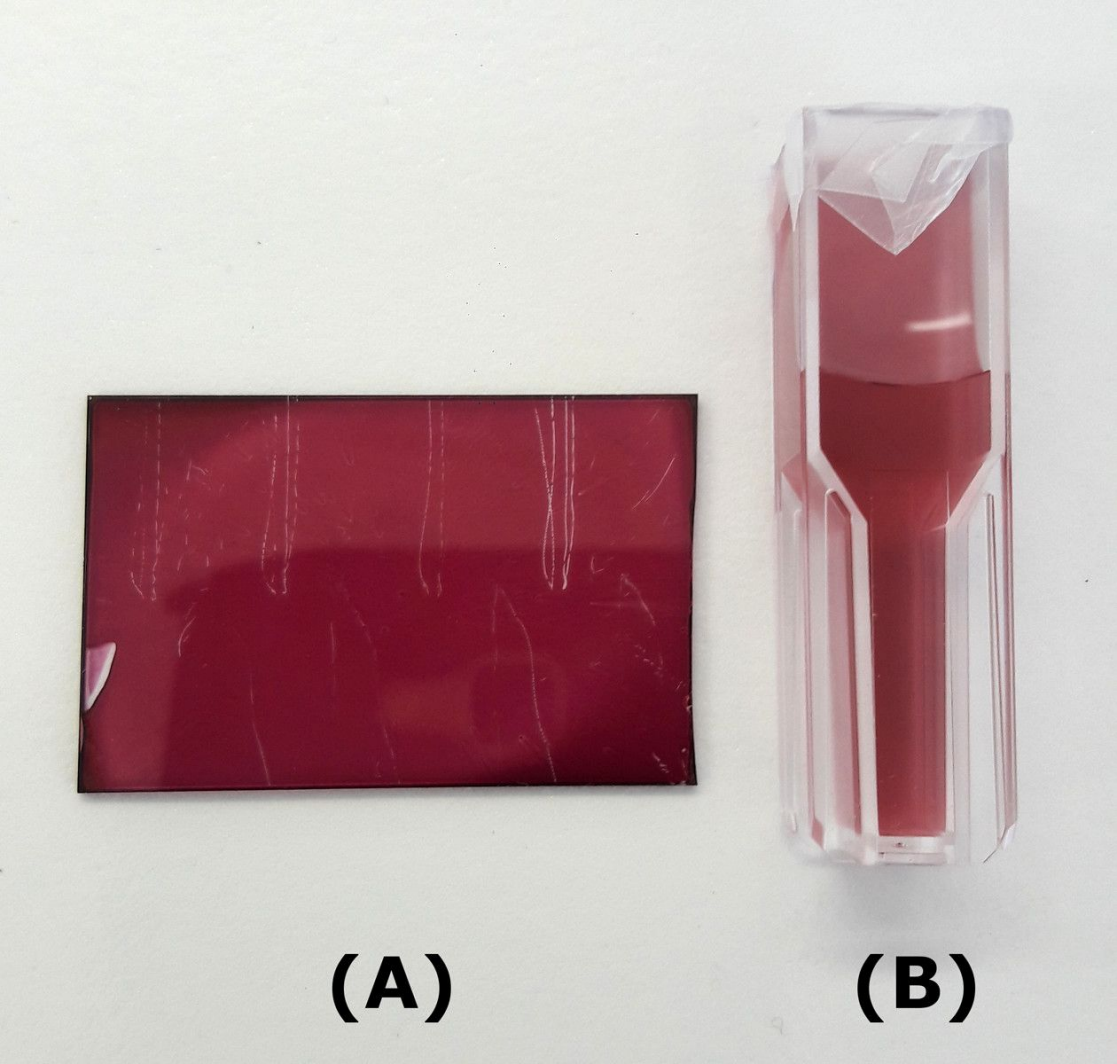
(A) Thin polymer film with in situ grown AuNPs (optical path: ca. 350 nm, [Au]/[PVA] mass ratio = 50%); (B) colloidal solution of AuNPs (optical path: 1 cm).

The optical properties of NPs come from collective oscillations of their conduction electrons excited by incident light. They are strongly influenced by shape and size of the particles but also by the dielectric properties of their environment, in particular when particles are embedded in a dielectric matrix. There are numerous synthesis methods for such nanocomposites, which globally belong to two categories: the synthesis of NPs in a liquid medium, which provides good control during their growth, or the in situ synthesis of NPs, e.g., by thermal annealing of a noble metal-doped solid phase such as HAuCl_4_-doped polymer films. In the latter approach there is less control over shape and the size of the NPs. In an isotropic film prepared from a homopolymer, we can expect the NPs to be spherical but their growth mechanisms are not yet completely understood and still subject to investigations. Nevertheless, this method, also known as one-pot synthesis, is simpler. In a single step, both the gold salt and the polymer are mixed to prepare the nanocomposite. The simplicity of the method is regarded as a great advantage for the production of nanocomposites for various applications in nano-optics. The principle of in situ methods is the use of one of the matrix components as a reducing agent for the metal salt. We therefore need a matrix that plays the role of reducing agent but also contributes to the stabilization of the NPs. Polymers like poly(vinylpyrrolidone) (PVP) or polyvinyl alcohol (PVA) are good candidates for this purpose. The self-stabilization process is another advantage of the in situ synthesis since it avoids the use of additional stabilizers such as citrate ions or sodium borohydride. The thermal annealing process allows for a reduction of the metal cations. Once in their neutral state, the metal atoms have to diffuse in the polymer matrix and to aggregate yielding nanoparticles. These steps are enhanced by annealing the films at a temperature higher than the glass transition temperature of the polymer. In practice, the in situ synthesis allowed us to obtain relatively thin films (300–500 nm) with an absorbance equivalent to that measured with a “classical” colloidal solution of AuNPs in a cuvette with 1 cm optical path (Figure 1). This means that the same absorbance can be obtained with a material thickness ca. 2·10^4^ times smaller. Passive Q-switches based on saturable absorption of AuNP-doped films are usually prepared in a two-steps consisting of synthesis and dispersion in the polymer matrix) [15]. Hence, it is important to investigate in detail potential advantages of a one-step synthesis. To the best of our knowledge, such an investigation has not been carried out yet.

In this article, we consider the characterization of backscattering of light from plasmonic nanocomposites doped with AuNPs, which is problematic for fiber optics integration, e.g. in Q-switched fiber lasers. This important issue has so far not been addressed. In particular, we investigate the modifications of the scattering properties of nanocomposites induced by the in situ growth of NPs. For this purpose, we performed the annealing of Au^3+^-doped PVA films and we quantified the scattering of light that is induced by the AuNP growth. We monitored the evolution of the locally resolved optical properties of the film using imaging ellipsometry (IE) and determined the roughness of the films using atomic force microscopy (AFM).

## Results and Discussion

### Optical scattering measurements

In preliminary experiments, Au-doped polymer films coated on glass were annealed in an oven at different temperatures (90–160 °C) over different periods of time (1–12 h). Different mass percentages of gold were also tested (1–3 wt %). At the end of the annealing, the films became reddish. The optical transmission was measured showing a plasmon resonance near 530 nm (data not shown). On the basis of these experiments, in order to achieve AuNP synthesis within reasonable time, 2% [Au]/[PVA] mass ratio and 135 °C were chosen as optimal experimental parameters. Similar results have been obtained by Sun and co-workers [20]. They systematically investigated the effect of the [Au]/[PVA] mass ratio and of the annealing temperature. Although the experiments have been carried out in a slightly different way, i.e., through the reducing the Au^3+^ ions with PVA in the liquid phase and casting the solution on substrates to obtain supported films, they provided us some interesting trends about the AuNP growth. The resonance position is not modified by the [Au]/[PVA] mass ratio, but when the mass ratio increases, the absorbance reaches an increasing plateau value. Moreover, increasing the annealing temperature increases the reduction kinetics. The plateau value is reached within shorter times. Besides playing a role in the growth kinetics of the nanoparticles by promoting the reduction of the Au^3+^ ions, the annealing temperature also modifies the polymer matrix itself. It induces cross-linking mainly by interconnecting hydroxy groups of the polymer chain. This cross-linking can occur at inter- or intramolecular levels [21–23].

**Figure 2:**
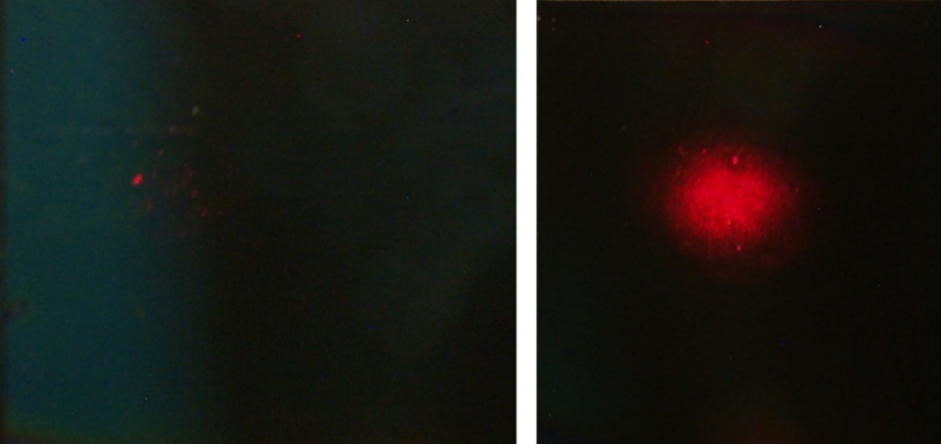
Optical scattering of an expanded laser beam (diameter 5 mm) on the sample. (Left) Undoped polymer film. (Right) Annealed PVA matrix embedding in situ synthesized AuNPs (incidence angle: 45°, observation angle: 0°, 12 mW HeNe laser at λ = 632.8 nm).

During annealing, it was also observed that the optical scattering of the laser beam strongly increased (Figure 2). This phenomenon can be related to the roughness parameter observed in AFM measurements as explained in the next subsection.

The most general way to characterize optically a surface is to measure its bi-directional reflection distribution function (BRDF). This function is wavelength-dependent and, at a given wavelength λ, it is defined by the ratio of the reflected irradiance to the incident irradiance:

[1]
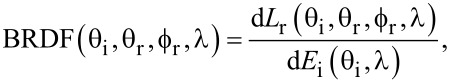


where θ_i_ and θ_r_ are the incidence and reflection angles, respectively, and ϕ_r_ is the azimuth angle. The reflected irradiance d*L*_r_ is directly measured by the instrument. The incident irradiance d*E*_i_ is measured in relation to the reflection characteristics of a commercial Spectralon^®^ reference surface :

[2]
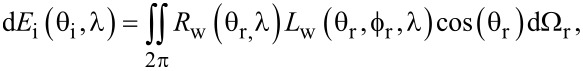


where dΩ_r_ is the elementary solid angle around the direction defined by θ_r_. The reflection coefficient of the Spectralon surface is *R*_w_≃ 0.99. *L*_w_ is the irradiance measured for that reference. Measuring the BRDF at one incidence angle therefore requires the measurement of the irradiance on two surfaces (sample and Spectralon).

**Figure 3:**
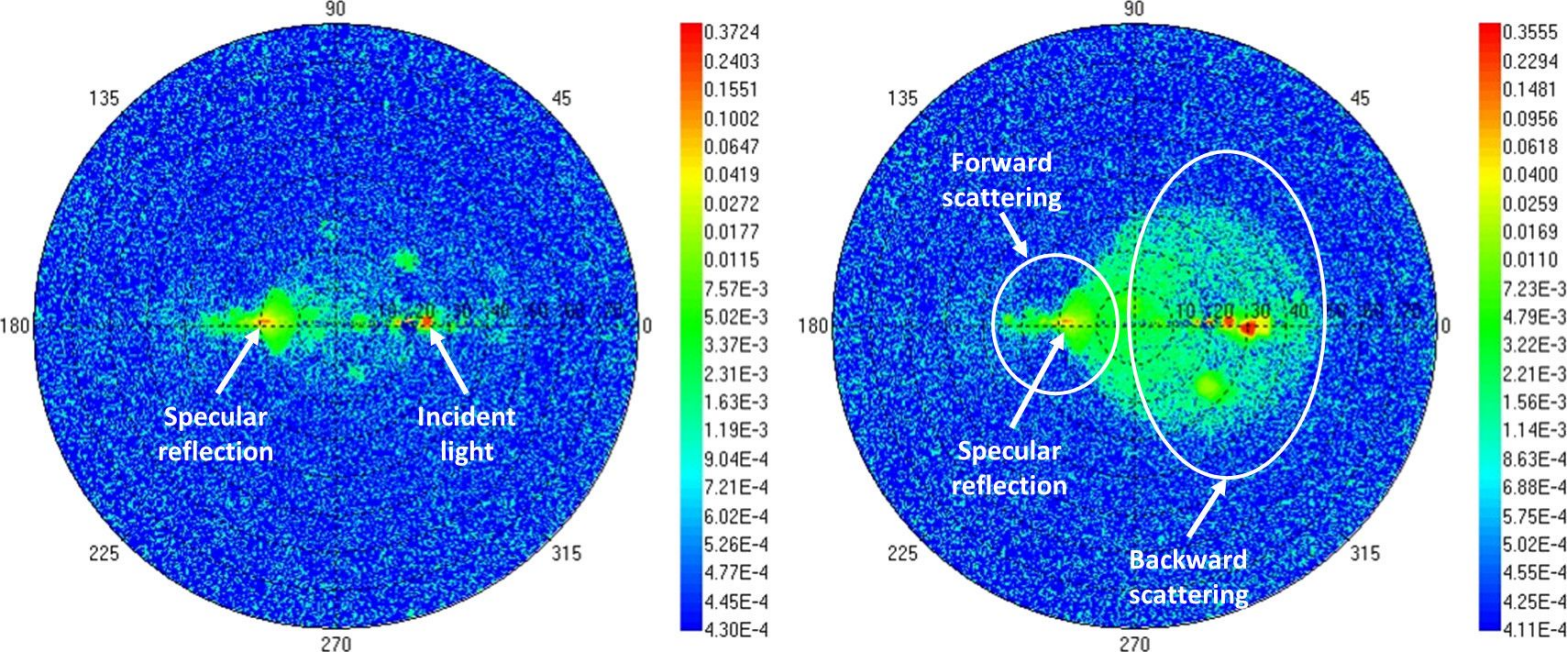
Experimental BRDF measurements at θ_i_ = −20°and λ = 570 nm. (Left) Non-annealed sample. (Right) Sample annealed 120 min at 135 °C. Data are presented on a logarithmic scale (arbitrary units). Specular reflection, forward and backward scattering regions are indicated in the plots. Dashed circles correspond to data measured at a given polar angle with respect to the normal of incidence (0–80°). Azimuth angles are varied along these circles (0–360°).

The BRDF was measured in collimated mode on samples before and after annealing. In both cases, the incidence angle θ_i_ was set to −20°. The wavelength was set to λ = 570 ± 10 nm, slightly off-resonance with the plasmon excitation at ca. 530 nm. The results are presented in Figure 3 on a logarithmic scale. Forward scattering and backscattering correspond, respectively, to the left and right parts of the circular plot. The incidence and scattered angles correspond to the values reported on the horizontal segment along the radius of the circle. The scattered azimuth angles correspond to the values reported along the outer circle. BRDF measurements show that the sample reflectance is neither isotropic nor specular. The scattering pattern considerably evolves with the annealing of the nanocomposite. Besides the specular reflection peak (at an angle of +20°), which is present for both samples, back-reflection is observed over a large area together with strong forward scattering out of the incidence plane.

**Figure 4:**
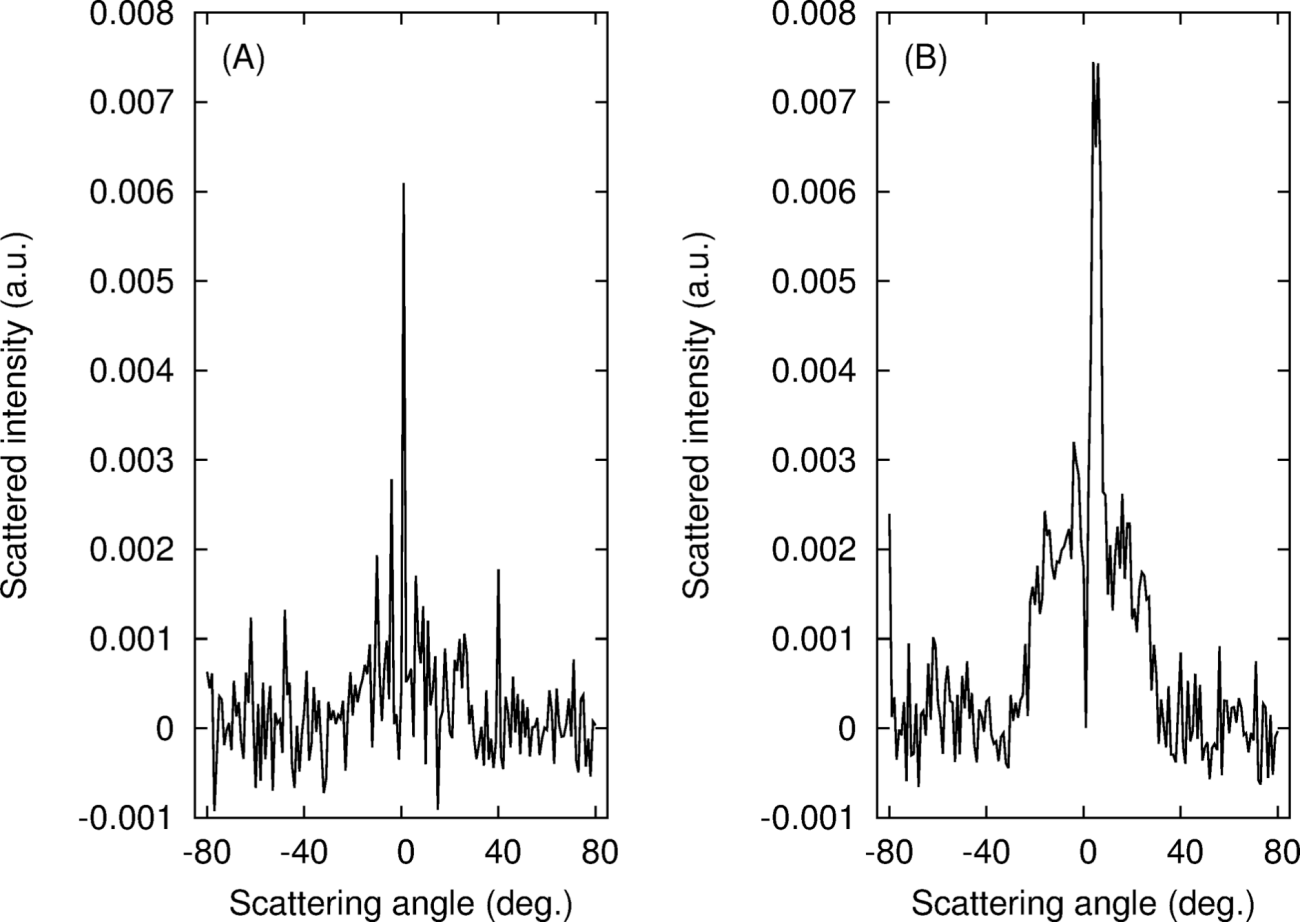
Scattered intensity in the plane orthogonal to the incidence plane (azimuth 90° or 270°) as a function of the scattering angle. (A) Non-annealed sample; (B) sample annealed for 120 min at 135 °C.

BRDF curves corresponding to cross sections of the previous measurements along the plane of azimuth ϕ_r_ = 90–270°, i.e., perpendicular to the incident plane, are presented in Figure 4 for the annealed and non-annealed samples. Even if the background is relatively noisy, the curves are clearly different from each other and the annealing process yields a well-defined scattering lobe at scattering angles between θ_r_ = −35° and θ_r_ = 35°. The scattered intensities are more or less symmetrical with respect to the incidence plane, θ*_r_* = 0°.

### Topographic surface characterization

**Figure 5:**
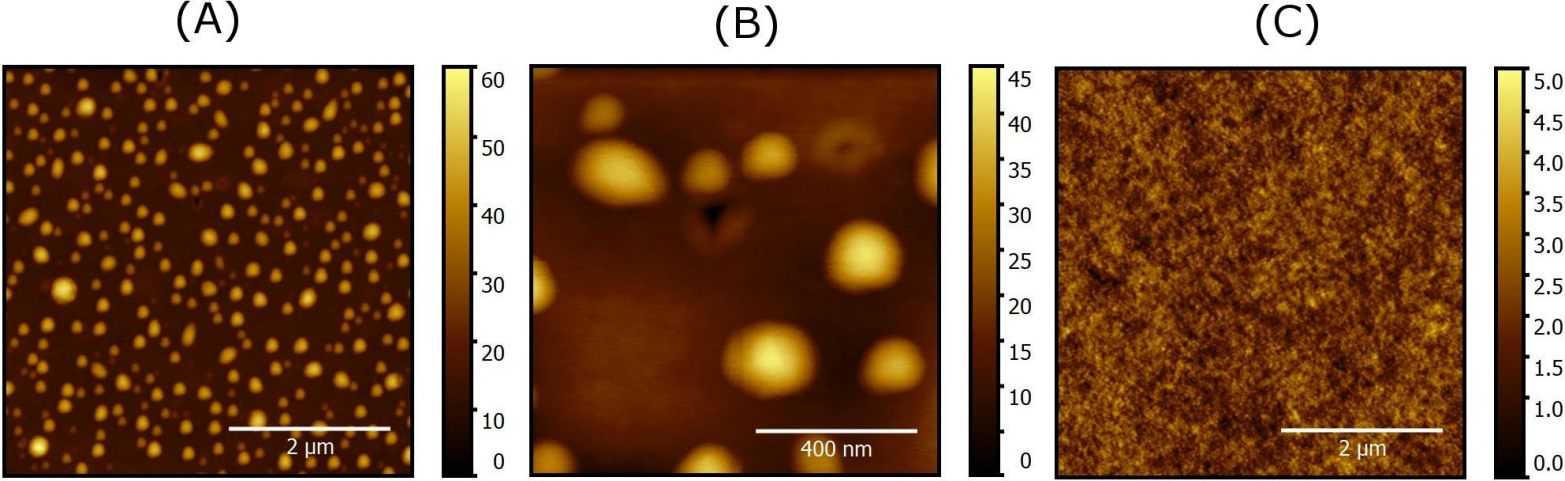
AFM topography images (Color scale bars in nanometers). (A) PVA matrix with embedded in situ synthesized AuNPs (image size: 5 μm × 5 μm). (B) Details of the previous image (image size: 1 μm × 1 μm)). (C) Undoped PVA matrix (image size: 5 μm × 5 μm).

The annealed gold nanocomposites were imaged by AFM. A typical image of 5 μm × 5 μm size is presented in Figure 5a. It unambiguously shows the presence of the AuNPs. The topography of the samples was characterized by the average surface roughness parameter (*S*_a_) and by the root-mean-square surface roughness parameter (*S*_q_). For the annealed sample, values of *S*_a_ = 6.28 nm and *S*_q_ = 8.22 nm were measured. Similar values were measured for the 10 μm × 10 μm images. These structural features do not appear on undoped films (Figure 5c). As typical roughness parameters for pure PVA films of corresponding thickness are *S*_a_ = 0.42 nm and *S*_q_ = 0.53 nm, i.e., about 10 to 12 times less, it is clear that the annealing process induces roughness at the air/polymer interface, which can be attributed to the in situ synthesis of the AuNPs.

**Figure 6:**
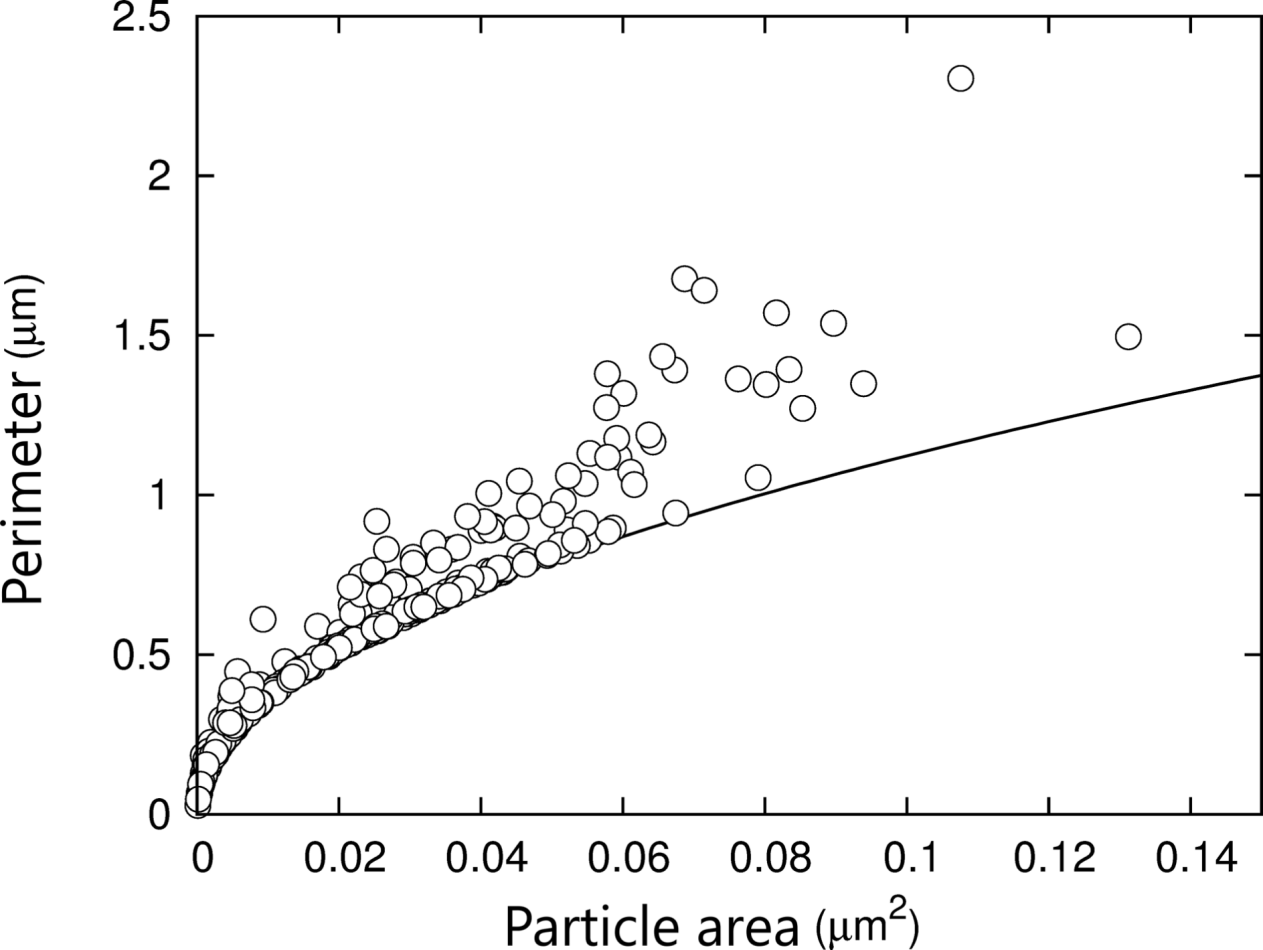
Perimeter–area relationship of the AuNPs. Symbols: experimental data determined by using a height-threshold method. Line: theoretical relationship (*P* = 2π*^1/2^A^1/2^*) between perimeter *P* and area *A* for circular AuNPs.

Besides spherical NPs, AuNPs with nearly triangular or hexagonal shapes are also observed (Figure 5b). Using a threshold method, nanoparticles can be isolated from the polymer matrix image and their shape/size characteristics can be determined (Figure 6). Similar morphological characteristics were also usually observed at a larger scale by optical microscopy and SEM [24]. Recently, the shape of the AuNPs was shown to be also dependent on the chemical nature of the counterions in the gold salt [25]. In the present samples, there is a deviation from the circular shape, for which the perimeter/area relationship is given by *P* = 2π*^1/2^A^1/2^* ≃ 3.55*A^1/2^* where *P* is the perimeter of the nanoparticle and *A* its projected area (Figure 6). The deviation from the latter relation reveals the presence of more complex nanoscale structures. In Figure 5a, the AuNPs occupy 31% of the surface. Nearest-neighbor distances between AuNPs were calculated and ranged from 98 to 406 nm with a mean value equal to 214 ± 54 nm. The data slightly differ from a normal distribution as confirmed by a Shapiro–Wilk statistical test results (*W* = 0.9838, p-value = 0.004583).

### Imaging ellipsometry

Imaging ellipsometry (IE) provides local information about the optical properties of a (multilayered) material. This local information is available at the micrometer-scale. Information is given by the ellipsometric angles Ψ and Δ, which are related to the ellipticity ρ defined as the ratio between the reflection coefficients of p- and s-polarisation :

[3]
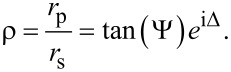


In a polarizer-compensator-sample-analyzer (PCSA) imaging ellipsometer, the polarizer angle (*P*) and the analyzer angle (*A*) are varied in order to achieve extinction of the signal. With a compensator angle C set to 45°, the angles *P*_min_ and *A*_min_ at which the extinction is observed are directly related to the ellipsometric angles Ψ and Δ through [26]:

[4]
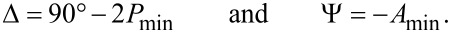


As the imaging ellipsometer is based on a nulling procedure, it does not provide direct information about the depolarization of the sample. Nevertheless, as the signal can almost be extinguished on the whole images, one may expect this to be negligible.

Ellipsometric data have to be processed according to an optical model to extract physical data. This model assumes flat interfaces between adjacent layers, each of them being characterized by its thickness and its (frequency-dependent) complex refractive index or dielectric function. In the case of layers containing mixed materials, which is the case for our nanocomposite layers, the optical properties of the components are combined according to mixing rules, the so-called “effective medium approximation” (EMA). For non-interacting nanoparticles embedded in a host matrix, Maxwell–Garnett EMA (MG-EMA) is usually used to interpret standard spectroscopic ellipsometric data. For metal volume fractions in the composite less than 1%, the effect of doping is usually not detected experimentally using MG-EMA when the optical measurements are carried out out-of-resonance. This effect has been modeled in Supporting Information File Supporting Information File 1. Moreover, going from standard ellipsometry to imaging ellipsometry, i.e., from a global optical response to a local response, brings down the spatial resolution to twice the wavelength of the incident light. The MG-EMA should therefore be used with caution and studied in deeper details. For those reasons, as the metal volume fraction used in our experiments is ca. 0.13%, our experimental data will be processed on the basis of a one-layer optical model with variable thickness and adjustable real refractive index.

**Figure 7:**
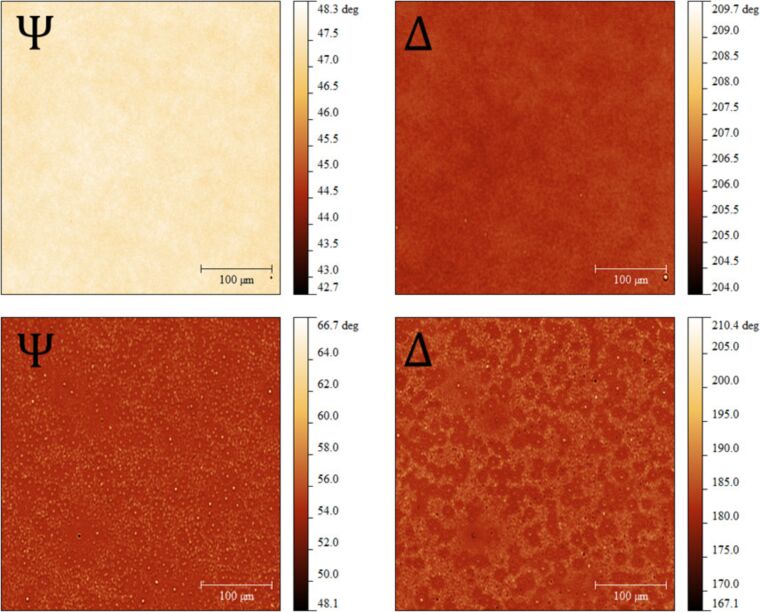
Maps of the ellipsometric angles Ψ and Δ of Au-doped PVA films. (Top) Before annealing. (Bottom) After annealing (135 °C, 120 min). (Left) Ψ angle. (Right) Δ angle. (Image size: 450 μm × 380 μm). Details of the annealed maps are given in Supporting Information File Supporting Information File 1 (Figure S4).

In our study, the IE optical response of Au-doped PVA films was recorded during annealing. The initial temperature was around 20 °C (room temperature). The temperature was raised up to 135 °C at a rate of 10 °C/min. Figure 7 shows maps of the ellipsometric angles Ψ and Δ recorded at the beginning and at the end of the growth on a 380 nm thick film. More Ψ and Δ maps corresponding to intermediate times are given in the Supporting Information File Supporting Information File 1 (Figure S1 and Figure S2). Before the annealing, the sample is clearly homogeneous (Figure 7, top images). After 120 min at 135 °C (Figure 7, bottom images), spots can be observed in the Ψ and Δ images. They are assumed to be the manifestation of light intensity diffraction patterns from the NPs despite the low volume fraction of gold in our samples (*f*_Au_ *<* 0.2%). Moreover, remembering that the angle Δ is related to the relative phase change undergone by the p- and s-polarized components of the incident light, the Δ maps clearly bring evidence for a local change of the relative phase upon reflection. As mentioned before, the surface roughness is quite small and we may therefore expect the effect of depolarization to be negligible. As shown by Fujiwara et al [27], AFM and ellipsometry measurements show the same trends when measuring the thickness of the roughness layer and the AFM roughness of thin films, although quantitative modeling is far more complex and requires, i.a., the correlation length of the surface to be taken into account [28]. So, the changes in Ψ and Δ are most likely due to the growth of the AuNPs, which locally change the optical response of the film.

Other causes of the local changes of the optical response such as temperature-induced thickness and refractive index change of the substrate can also be neglected. The thermal expansion coefficient of silicon oxide (0.24 × 10^−6^ K^−1^) and its thermo-optic coefficient d*n*/d*T* (1.29 × 10^−5^ K^−1^) [29–30] lead to statistically insignificant effects on the optical response when the sample temperature is increased from room temperature to 135 °C. A similar behavior is expected for the polymer matrix with values of ca. 1 × 10^−4^ K^−1^ for the linear thermal expansion coefficient and a negative value of ca. −10^−4^ K^−1^ for d*n*/d*T* [31].

**Figure 8:**
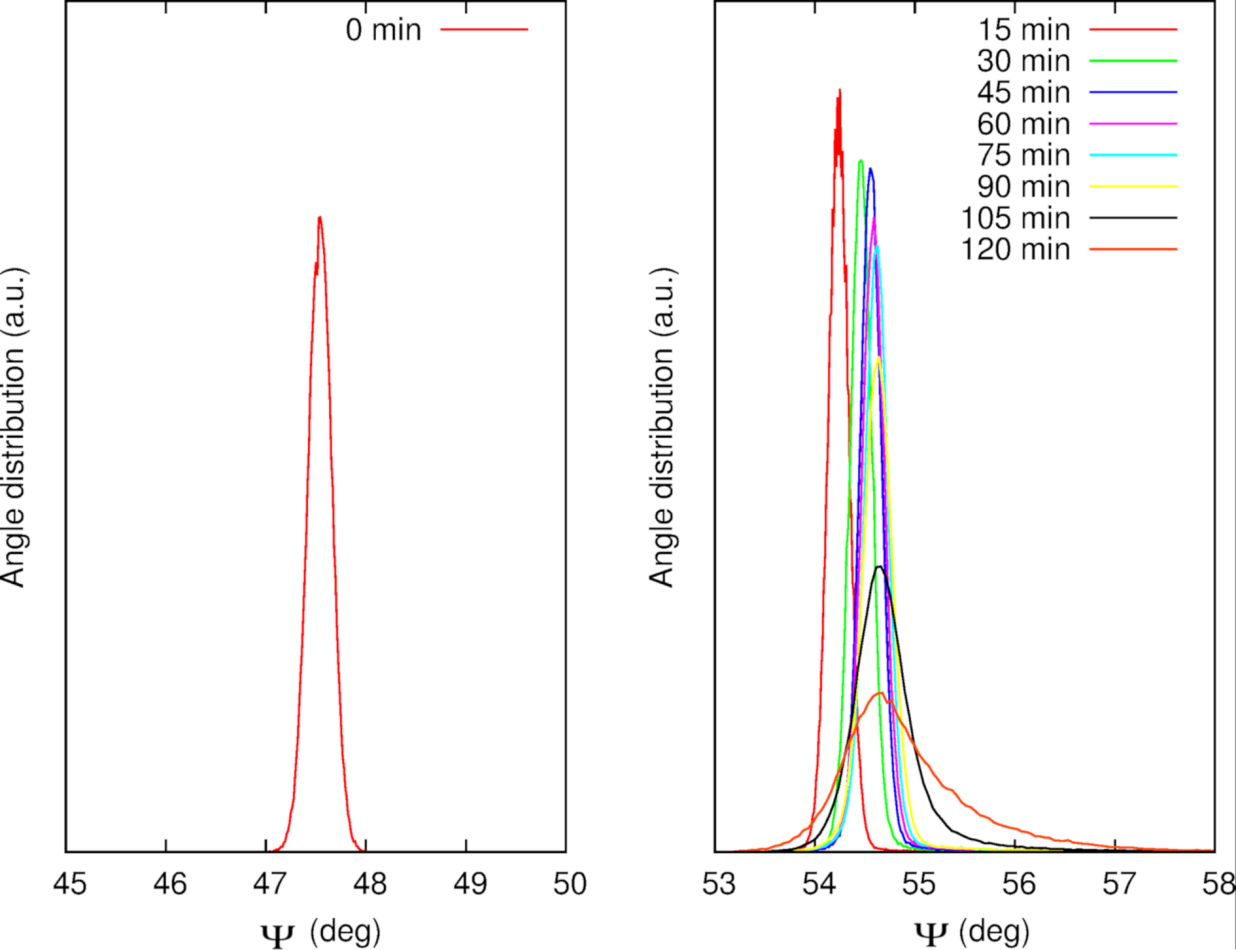
Statistical distributions of the Ψ angles. (Left) Before annealing. (Right) As function of the annealing time.

**Figure 9:**
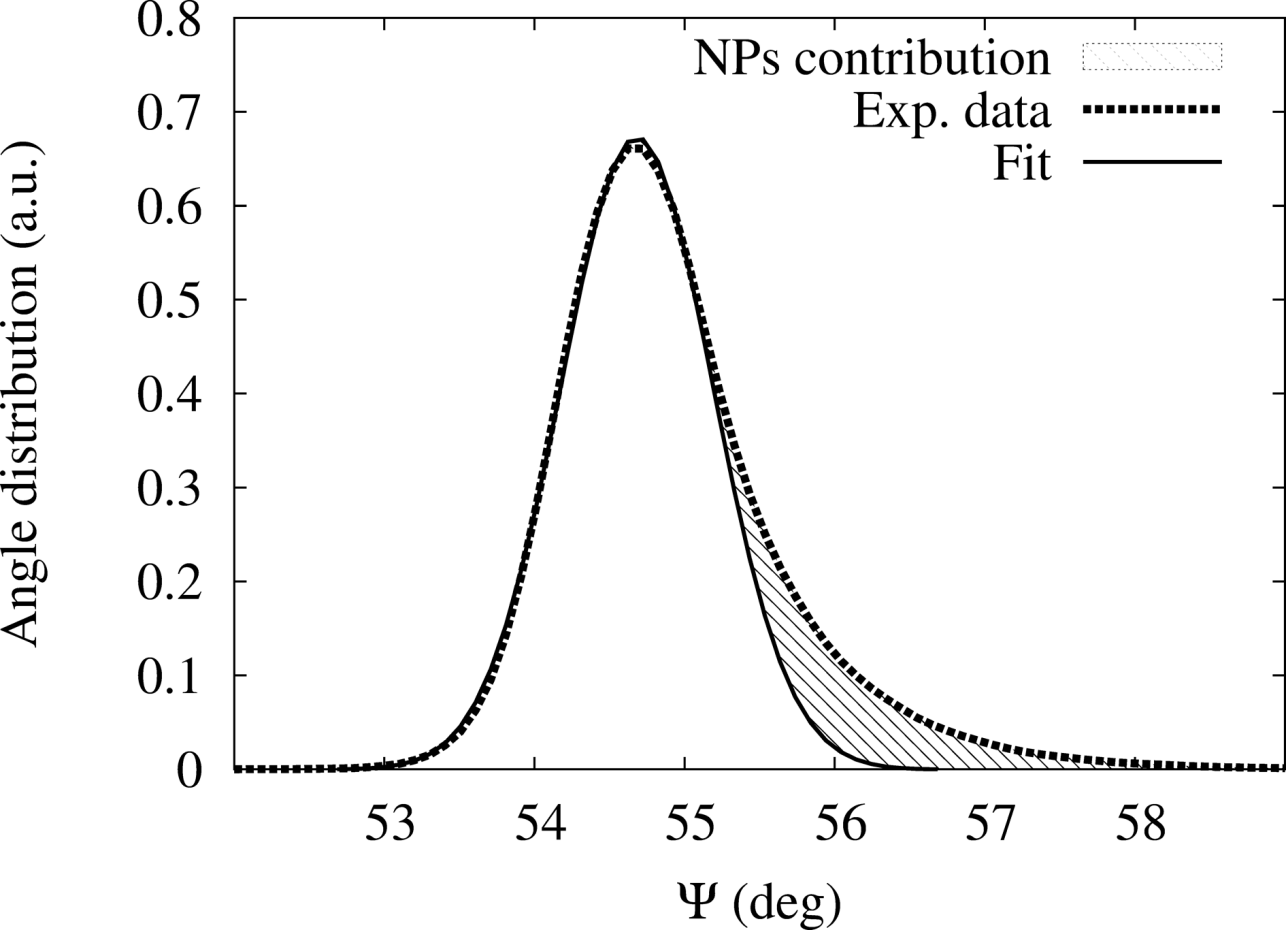
Statistical distribution of the Ψ angles after annealing (*t* = 120 min). Dashed line: experimental data; solid line: Gaussian fit using the most left part of the data; shaded area: contribution of the AuNPs.

Statistical distributions of the ellipsometric angle Ψ as a function of the annealing time were studied for the sample corresponding to the previous figures (Figure 8). Initially, the values follow a symmetric distribution around 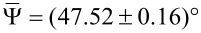 (Figure 8, left panel). During annealing, the distribution shifts towards higher values while its shape starts showing an increasing tail on the right, which is attributed to the growth of the AuNPs (Figure 8, right panel). As a consequence of the broadening, the height of the peak strongly decreases. Similar results are obtained for the angle Δ (data not shown). The statistical distribution of Ψ, which is symmetric at the beginning of the annealing (*t* = 0, 5, 10 min), clearly becomes asymmetric with a marked contribution of the AuNPs to the response at higher Ψ values as the annealing proceeds. This is illustrated in Figure 9 at the end of the annealing (*t* = 120 min) where the experimental distribution of Ψ is compared to a Gaussian fit of the lower experimental Ψ values. The deviation from the gaussian fit corresponds to the contributions of the AuNPs growth and is represented by the shaded area.

**Figure 10:**
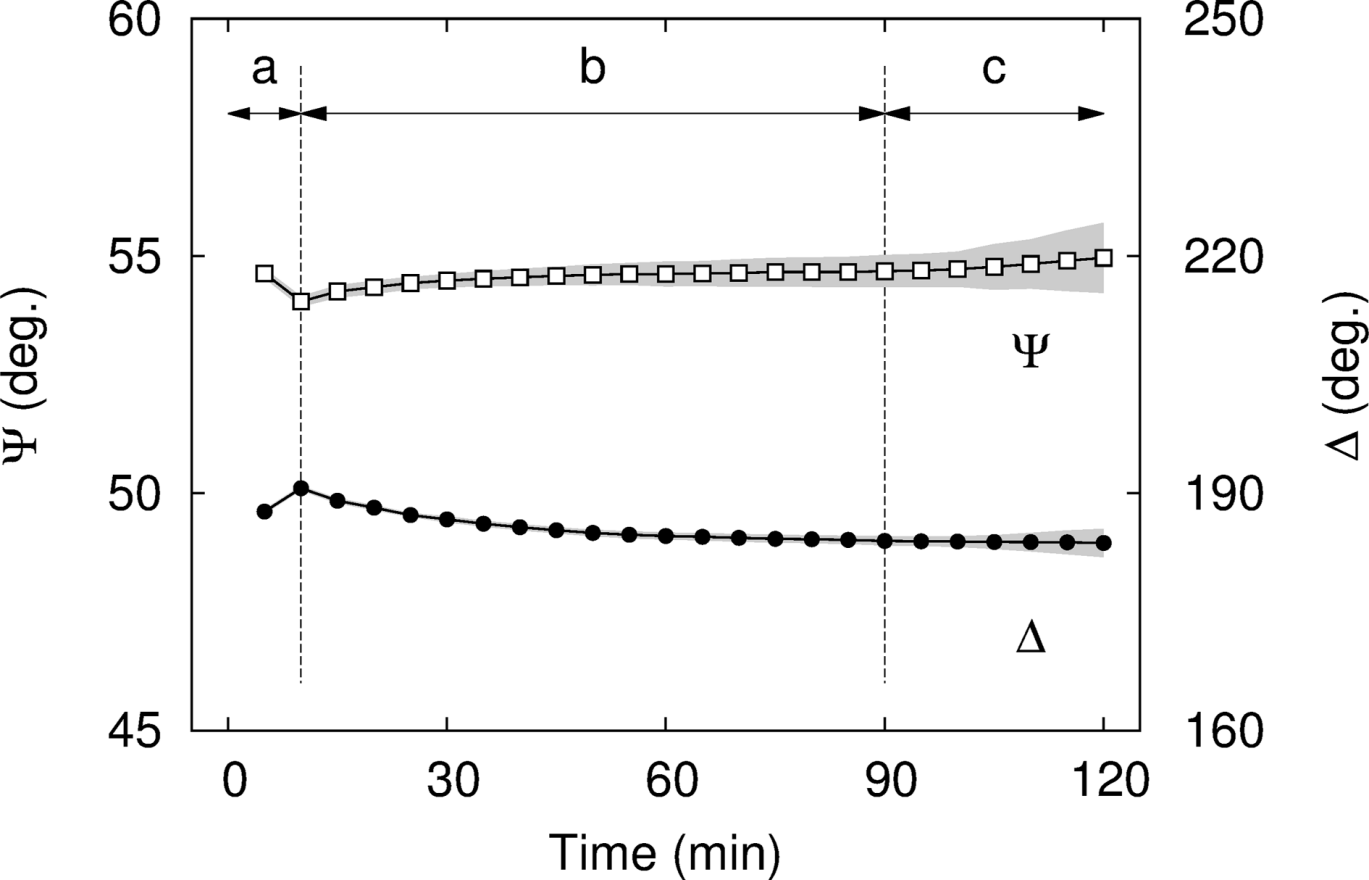
Time evolution of the mean values of ellipsometric angles during annealing. Open squares: Ψ; closed circles: Δ; shaded area: standard deviations. The zones (a), (b), and (c) refer to the evaporation of residual solvent, to the relaxation of thickness and to the changes of the refractive index, respectively.

**Figure 11:**
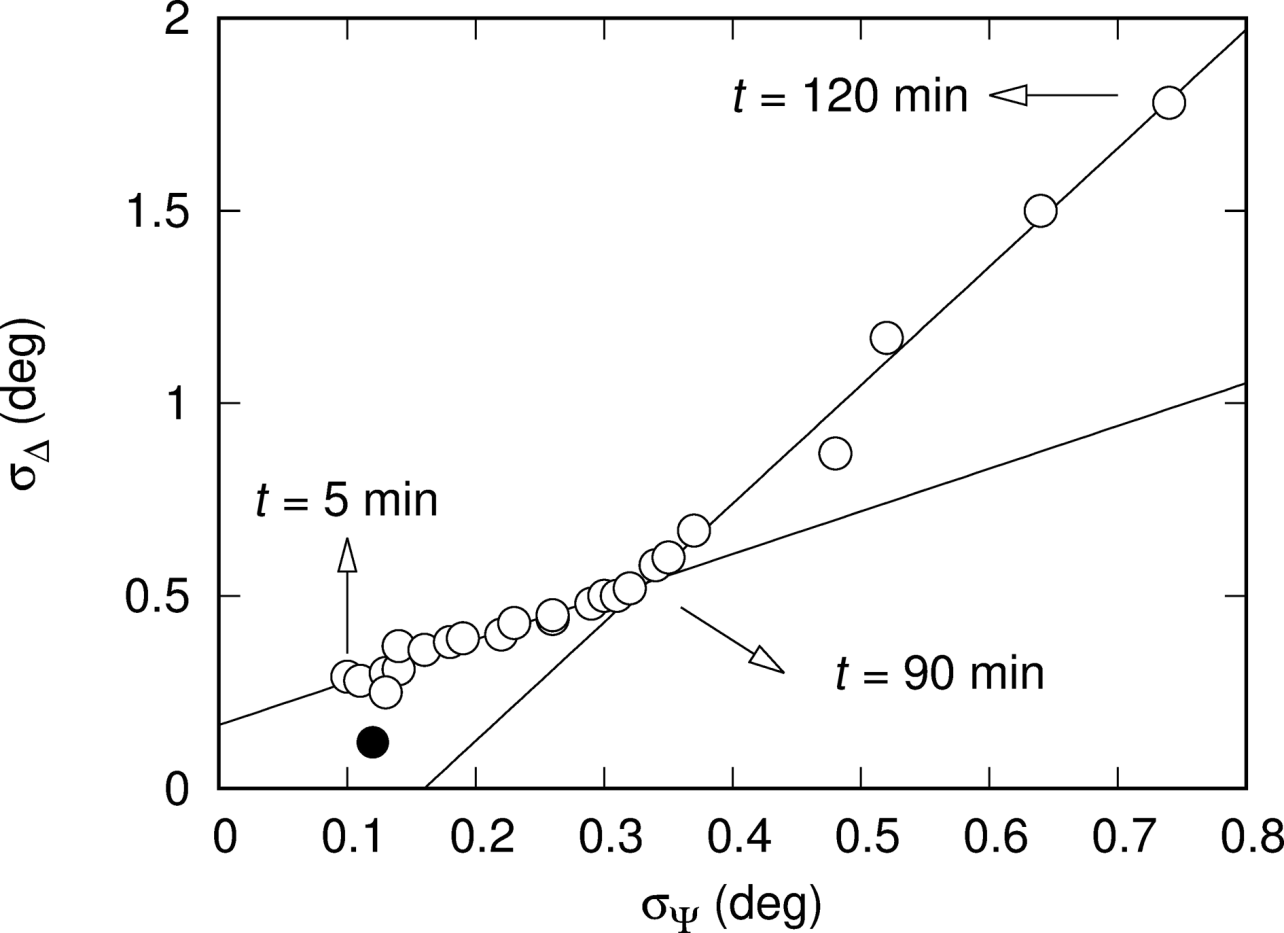
Correlation plot between the standard deviation of the ellipsometric angles Ψ and Δ during film annealing (open circles: experimental data, lines: linear fits). The filled circle corresponds to *t* = 0 min.

The mean values of Ψ and Δ are reported in Figure 10 as functions of the time. The behavior of the sample is complex during the first 10 min of annealing. During that period, the temperature rises from room temperature to 135 °C. We expect the mechanical constraints induced by the spin-coating to relax as soon as the sample temperature reaches *T*_g_, the glass transition temperature of the polymer. For bulk PVA, *T*_g_ equals 85 °C [32]. As already reported for other polymers, *T*_g_ is also a function of the film thickness and differs from the bulk value of *T*_g_ [33]. It can also be modified by doping. Since the pioneering work of Keddie and co-workers [34], it is well known that the ellipsometric response of films changes when going through the glass transition. After this initial period (zone (a) in Figure 10), the values stabilize and only undergo slow variations (zone (b) in Figure 10). The sample then becomes heterogeneous due to the growth of nanoparticles, as shown by the increase of the standard deviation of the measurements (zone (c) in Figure 10). The evolution of the standard deviations of Ψ and Δ during the annealing is presented in Figure 11. There are clearly two regimes in the dynamics: the first part, before 90 min, corresponding to a slow variation of the standard deviations, and the second part, after 90 min, with a large increase of the standard deviations of both angles corresponding to the growth of the AuNPs. In the dynamics, consecutive data point are separated by Δ*t* = 5 min.

**Figure 12:**
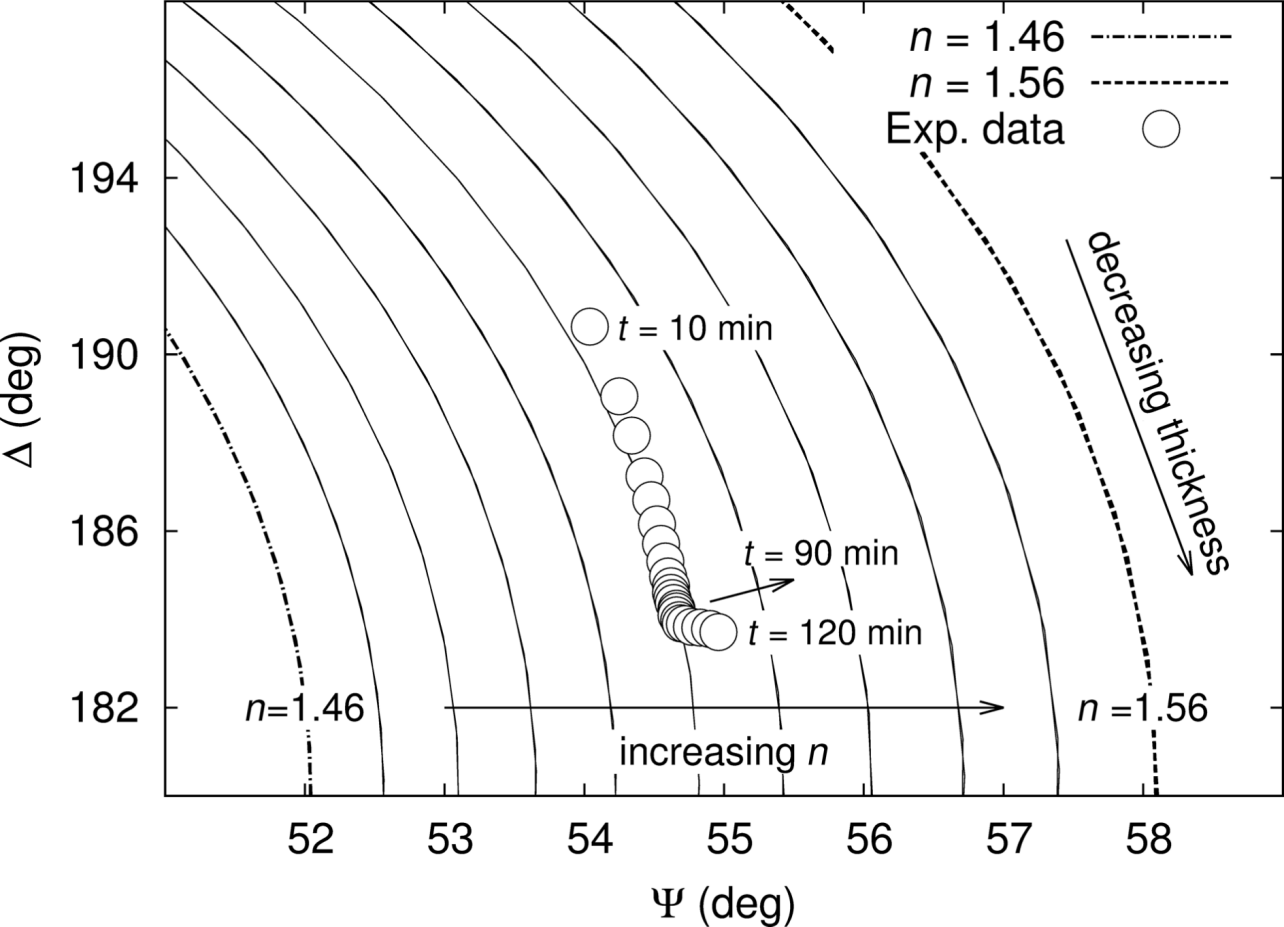
Constant angle of incidence Ψ–Δ curves at the wavelength λ = 658 nm and the angle of incidence θ_i_ = 42° for refractive indices varying from *n* = 1.46 (left-most curve) to *n* = 1.56 (right-most curve) and varying film thickness. Open circles: ellipsometric response of the Au–PVA nanocomposite during annealing (time labels added to identify the time course).

The information of the two graphs in Figure 10 can be combined on a single curve in (Ψ, Δ)-space that can be superimposed on theoretical curves of constant angle of incidence (CAI) calculated at θ_i_ = 42°and fixed wavelength (λ = 658 nm) [26]. These curves can be parametrized as a function of the film thickness and of the refractive index. As the operating wavelength of the ellipsometer is off-resonance with respect to the localized plasmon wavelength (λ_SPR_ ≃ 530 nm) and the volume fraction of gold in the sample is low (*f*_Au_ *<* 0.20%), the extinction coefficient of the film can be assumed to be small and therefore neglectable. Theoretical calculations were made using the Maxwell–Garnett effective medium approximation (MG-EMA) for spherical nanoparticles (Figure S3, Supporting Information File Supporting Information File 1). They showed that, at the operating wavelength of the ellipsometer and at the doping levels considered in this study (*f*_Au_ ≤ 0.3% v/v), only the refractive index is expected to slightly vary according to the gold concentrations, and that the change in the extinction coefficient would be within the error bars of the ellipsometric measurements. In Figure 12, the refractive index of the CAI curves increases from *n* = 1.46 to *n* = 1.56 (left to right). The direction of the variation of the film thickness among these curves is also indicated by an arrow. The experimental data corresponding to the slow variation of the ellipsometric angles clearly belong to two different regimes. The first part of the dynamics (*t* = 0–90 min) corresponds to a decrease of the film thickness (approximately from 370 nm after 10 min of annealing to 365 nm after 90 min of annealing). The second part (*t* = 90–120 min), associated to the increase of the standard deviation of the data, corresponds to a local increase of the refractive index. As mentioned previously, the thermo-optic coefficient of pure PVA is negative, inducing a decrease of refractive index when temperature increases. Thus, this local increase of the refractive index during the second part of the dynamic is due to the growth of the AuNPs, and not the thermal response of the PVA film. Let us briefly come back to the concept of off-resonance detection of the plasmonic response. Different approaches are possible to process the ellipsometric data related to plasmonic nanocomposites. Among them, the use of classical oscillators to directly describe the localized plasmon resonance in the global complex dielectric function of the material ε_tot_ is widely used. In this case, ε_tot_ is the sum of two contributions, namely the matrix and a Lorentzian oscillator describing the resonance. This Kramers–Kronig consistent approach learns us that the spectral range over which the real part of the dielectric function varies is larger than the one over which the energy absorption takes place. When far off the resonance, detecting the optical response of the growing metal nanoparticles can therefore be achieved in considering the variations of the real part of the refractive index.

## Conclusion

We studied the optical scattering of nanocomposites containing in situ grown AuNPs. The work focused on the optical response of Au-doped PVA films during annealing. The increase of the surface roughness due to the growth of NPs, as observed by AFM, was confirmed by BRDF measurements, which were symmetric with respect to the plane of incidence and showed strong backscattering. Thanks to imaging ellipsometry, we clearly observed diffraction patterns due to the formation of NPs in spite of the low volume fraction of gold in the nanocomposite and of the nanometric size of the AuNPs. Superimposition of ellipsometric measurements recorded during the annealing and theoretical Ψ–Δ curves allowed us to decompose the dynamics in two parts. The first part, at the early stage of annealing, corresponds to a decrease of film thickness while the second part was attributed to an increase of the refractive index. Our observation that the in situ synthesis of NPs produces some roughness at the film surface and hence backscattering, is of importance for the integration of such Au nanocomposite films in Q-switched fiber lasers.

## Experimental

### Chemicals

HAuCl_4_ and poly(vinyl alcohol) (PVA, 87–89% hydrolyzed, *M*_W_ 13000–23000) were purchased from Sigma-Aldrich and used as received. All syntheses were carried out using 18 MΩ·cm Milli-Q water.

### Nanocomposite preparation

The in situ synthesis method is based on the one used in previous studies of Voué and co-workers [35–36] to prepare Ag–PVA films. The initial experimental protocol for Ag–PVA nanocomposites is that of Porel and co-workers [37]. With respect to the experimental protocols presented in [35–36], the main changes are a) the use of a gold salt and of a PVA with lower molecular weight and b) the spinning conditions (lower speed but longer times). All these changes resulted in the deposition of thinner films doped with Au^+3^ ions.

Briefly, in a typical synthesis, a 10 wt % PVA stock solution was prepared by heating the required amount of PVA at 85 °C under reflux till complete dissolution of the polymer. After cooling the solution to room temperature, 0.1 M HAuCl_4_ solution and water were added to the polymer solution to obtain a 8% polymer solution containing 2% of gold. The latter percentage refers to the [Au]/[PVA] mass ratio. After mixing and filtrating the solution on a 0.45 μm Millipore filter, the solution containing polymer and Au^3+^ cations was spin-coated using a Laurell WS-650-23B coater on RCA-cleaned Si(100) wafers fragments (2000 rpm, 90 s). Based on an average volume mass for PVA of 1.26 g/cm^3^, the 2% gold-to-PVA mass ratio corresponds to a volume fraction of *f*_Au_ = 0.13% in the dry film.

### Optical scattering measurements

Optical scattering of the nanocomposite films was analyzed by calculating the bi-directional reflection distribution function (BRDF). They BRDF was calculated from measurements of radiance with a EZ Contrast XL80MS scatterometer (ELDIM, France). Principle and operating mode have been described in Bay and co-workers [38]. Briefly, the experimental setup is a system of Fourier lenses. It redirects all light rays emitted by the sample along all emergence directions in such a way that they form an image of the source in the Fourier plane. A polar angle and an azimuth angle characterizing each emergence direction constitute a set of coordinates for the emergence directions. Each direction is mapped to a point on a disk on the Fourier plane, before the image is transferred to a CCD sensor. The sensor measures therefore all the intensities in the disk-shaped map, which contains a planar projection of the intensity distribution in the emergence hemisphere. The wavelength is chosen by rotating colored filters, covering the visible region. The ranges covered for the azimuth angles and the polar angles are 2π and 0–80°, respectively. In our experiments, the radiance was measured in collimated mode (λ = 570 ± 10 nm) with an angle of incidence (AOI) of θ_i_ = −20° (note that incidence angles are conventionally negative in BRDF measurements). A 12 mW HeNe Laser was also used in preliminary experiments in order to qualitatively evidence scattering after the annealing of the samples.

### Atomic force microscopy

The topography of the films was studied with a Park XE70 AFM (Park Systems Corp., Korea), operated in air in intermittent-contact mode with commercial ACTA tips (resonance frequency 309 kHz). Areas of 5 μm × 5 μm, 2 μm × 2 μm and 1 μm × 1 μm were imaged for every sample. The resolution of the images was 256 pixels × 256 pixels. The data were processed with the Gwyddion software (http://gwyddion.net/) [39].

### Imaging ellipsometry

Imaging ellipsometry (IE) measurements were carried out using an EP3 single-wavelength imaging ellipsometer operating at 658 nm (Accurion GmbH, Göttingen, Germany). Ellipsometric angle (Ψ and Δ) images were recorded at an angle of incidence θ_i_ of 42°. The lateral resolution was about 1 μm/pixel and the mapped surfaces were 450 μm × 380 μm large. The ellipsometric data were processed either with the EP4 software (Accurion) or using custom-written routines. Ellipsometric images were taken during the annealing every 5 min during the whole temperature ramp and stabilization period.

### Annealing

Annealing of the samples was carried out using a THMS600 Linkam heating/cooling stage. In a typical annealing experiment, a temperature ramp of 10 °C/min starting at room temperature was applied. When the temperature reached 135 °C, it was stabilized for 90 or 120 min. Thermal fluctuations were less than 0.1 °C.

## Supporting Information

In the Supporting Information, more Ψ and Δ ellipsometric images (Figure S1 and Figure S2) recorded during the annealing of a 380 nm thick Au^3+^-doped PVA film (doping level 2% w/w) are given. Theoretical optical properties of Au–PVA nanocomposites are given in Figure S3. The properties were calculated using the Maxwell–Garnett approximation, assuming a spherical shape for the NPs (depolarisation factor equal to 1/3). Details of the Ψ and Δ maps after annealing in Figure 7 (bottom images) are given in Figure S4.

File 1 Additional experimental data.
