# Supplementary material for: Growth dynamics and light scattering of gold nanoparticles in situ synthesized at high concentration in thin polymer films
Source: Beilstein J Nanotechnol. 2019 Aug 23;10:1768–77. doi: 10.3762/bjnano.10.172 (PMC6720325; doi:10.3762/bjnano.10.172)
Supplement: File 1 — Additional experimental data. [file Beilstein_J_Nanotechnol-10-1768-s001.pdf]

BEILSTEIN JOURNAL OF NANOTECHNOLOGY

Supporting Information

for

Growth dynamics and light scattering of gold nanoparticles in situ synthesized at high concentration in thin polymer films

Corentin Guyot, Philippe Vandestrick, Ingrid Marenne, Olivier Deparis and Michel Voué

Beilstein J. Nanotechnol. **2019,** *10,* 1768–1777. doi:10.3762/bjnano.10.172

Additional experimental data

License and Terms: This is a supporting information file under the terms of the Creative Commons Attribution License (http://creativecommons.org/licenses/by/4.0). Please note that the reuse, redistribution and reproduction in particular requires that the authors and source are credited.
The license is subject to the *Beilstein Journal of Nanotechnology* terms and conditions: (https://www.beilstein-journals.org/bjnano)

Figure S1 and Figure S2 show the Δ and Ψ ellipsometric images recorded during the annealing of a 380 nm thick Au^{3+}-doped PVA film (doping level: 2% w/w). The annealing time increases from left to right and from top to bottom with a step of 15 min between each image. The temperature is maintained at 135 °C, except for the first image for which T = 31 °C. Before annealing, the sample is clearly homogeneous (top images). After 120 min at 135 °C(bottom images), spots can be observed in the Ψ and Δ images showing at the macroscopic level the effect of the NPs growth in the polymer matrix.

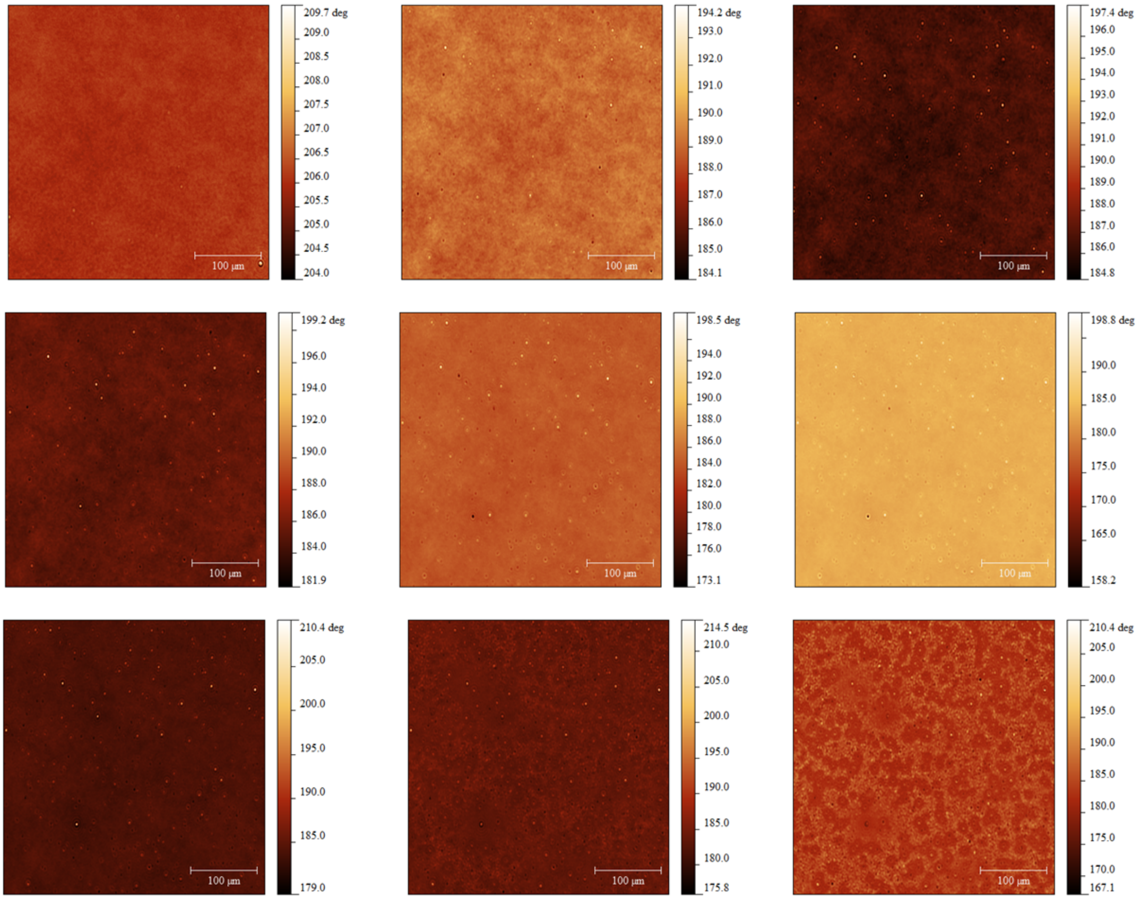

Figure S1: Δ maps of Au-doped PVA films (Image size: 450 µm × 380 µm). Annealing time increases from left to right and from top to bottom: t = 0, 15, 30, 45, 60, 75, 90, 105, 120 min. Temperature is maintained at 135 °C, except for the first image for which T = 31 °C.

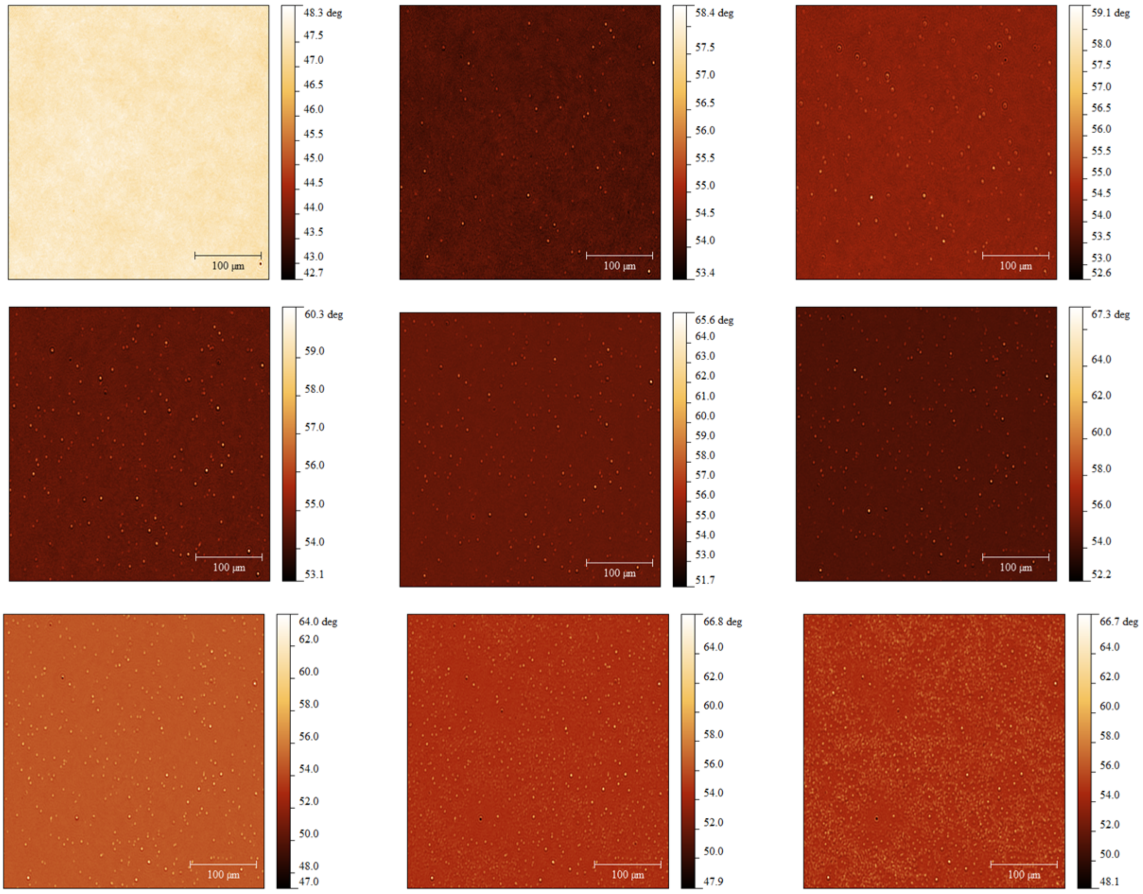

Figure S2: Ψ maps of Au-doped PVA films (Image size: 450 µm × 380 µm). Same experimental conditions as in Figure S1.

Theoretical optical properties of Au–PVA nanocomposites are given in Figure S3. The properties were calculated using the Maxwell–Garnett approximation. The gold particles were considered to be spherical and therefore the depolarization factor was set to 1/3. Optical properties of gold were taken from Palik's handbook of optical constants [1]. Optical properties of the pure PVA films were obtained by fitting the spectroscopic ellipsometry response by a Cauchy dispersion law for $n(\lambda) = A + B/\lambda^2$ [2], yielding a refractive index of undoped PVA equal to n = 1.511 at 658 nm. It increases to 1.518 and 1.525 when the volume fraction of gold equals 0.15% and 0.30%, respectively. In the meanwhile, the extinction coefficient increases from 7×10^{-4} to 1.4×10^{-3}. From this, it can be

seen that, at 658 nm, only the increase of the refractive index can be measured, the increase of the extinction coefficient being within the error bars of the ellipsometric measurement.

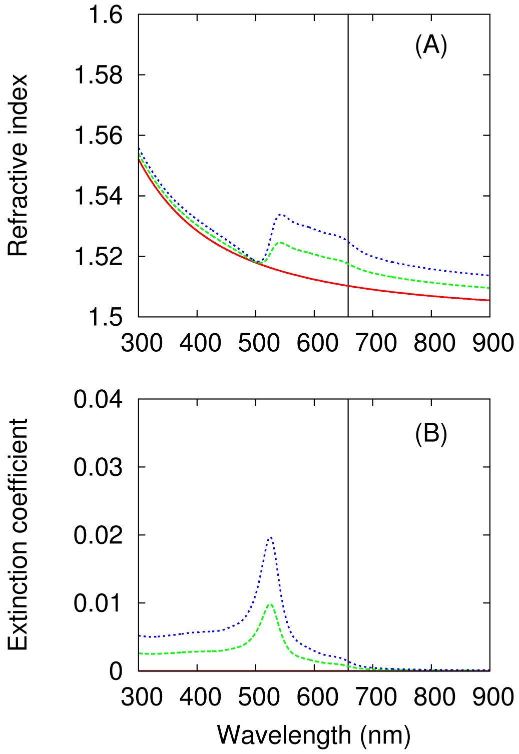

Figure S3: Optical properties of Au–PVA nanocomposites. (A) Refractive index, (B) extinction coefficient. (Red plain line: pure PVA, dashed green line: Au–PVA with a 0.15% Au volume fraction, dashed blue line: same with volume fraction of 0.30%, vertical black line: operating wavelength of the imaging ellipsometer).

Details of Figure 7 of the main manuscript are presented in Figure S4. It clearly shows that different zones surrounding the NPs are present in the Δ image, while less evident in the Ψ image. This supports the hypothesis of metal NP growth, the primary effect of which is the local modification of the relative phase between the *p*- and the *s*-components of the impinging light, i.e., the ellipsometric angle Δ. Under our experimental conditions, it appears that Δ is more sensitive to the local optical response when it remains low to moderate. Indeed, Ψ is closely related to the intensity changes of the reflected light, as measured in a conventional transmission or reflection UV–vis spectrophotometry with polarized light. The growth of the NPs requires the diffusion of metal atoms through the

interlaced network of the polymer chains. This diffusion is facilitated by the annealing the samples above T_g and the larger NPs, i.e., only those the effect of which can be detected in the optical response, form by some kind of drainage of the metal atoms in their neighborhood, leaving a depletion zone around the NPs.

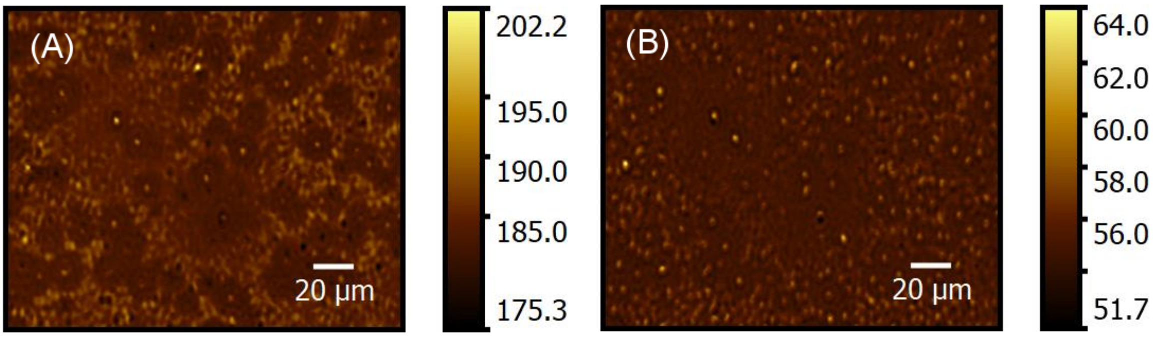

Figure S4: Details of Figure 7 of the main manuscript (after annealing): (A) Δ, (B) Ψ

References

1. *Handbook of Optical Constants of Solids, Vol. 1*; Palik, E. D., Ed.; Academic Press, 1997; doi:10.1016/B978-012544415-6.50004-2.

2. *Handbook of Ellipsometry*; Tompkins, H. G., Irene, E. A., Eds.; Springer-Verlag GmbH & Co. KG: Heidelberg, Germany, 2005; doi:10.1016/B978-0-8155-1499-2.50016-2.
